# The Role of Daily Steps in the Treatment of Major Depressive Disorder: Secondary Analysis of a Randomized Controlled Trial of a 6-Month Internet-Based, Mindfulness-Based Cognitive Behavioral Therapy Intervention for Youth

**DOI:** 10.2196/46419

**Published:** 2023-12-08

**Authors:** Kevin Dang, Paul Ritvo, Joel Katz, David Gratzer, Yuliya Knyahnytska, Abigail Ortiz, Clarice Walters, Mohamed Attia, Christina Gonzalez-Torres, Andrew Lustig, Zafiris Daskalakis

**Affiliations:** 1 School of Kinesiology and Health Sciences York University Toronto, ON Canada; 2 Department of Psychology York University Toronto, ON Canada; 3 Centre for Addiction and Mental Health Toronto, ON Canada; 4 Department of Psychiatry University of Toronto Toronto, ON Canada; 5 Department of Psychiatry University of California San Diego San Diego, CA United States

**Keywords:** accelerometer, anxiety, CBT, chronic pain, cognitive behavioral therapy, controlled trials, depression, depressive symptoms, digital health, eHealth, exercise, fitbit, intervention study, longitudinal study, major depressive disorder, mHealth, mindfulness, mindfulness-based CBT, objectively measured activity, online health, online intervention, online therapy, pain, physical activity, prospective study, randomized controlled trial, RCT, step, steps

## Abstract

**Background:**

Current evidence supports physical activity (PA) as an adjunctive treatment for major depressive disorder (MDD). Few studies, however, have examined the relationship between objectively measured PA and MDD treatment outcomes using prospective data.

**Objective:**

This study is a secondary analysis of data from a 24-week internet-based, mindfulness-based cognitive behavioral therapy program for MDD. The purpose of this analysis was twofold: (1) to examine average daily step counts in relation to MDD symptom improvement, and whether pain moderated this relationship; and (2) to examine whether changes in step activity (ie, step trajectories) during treatment were associated with baseline symptoms and symptom improvement.

**Methods:**

Patients from the Centre for Addiction and Mental Health were part of a randomized controlled trial evaluating the effects of internet-based, mindfulness-based cognitive behavioral therapy for young adults (aged 18-30 years old) with MDD. Data from 20 participants who had completed the intervention were analyzed. PA, in the form of objectively measured steps, was measured using the Fitbit-HR Charge 2 (Fitbit Inc), and self-reported depression severity was measured with the Beck Depression Inventory-II (BDI-II). Linear regression analysis was used to test PA’s relationship with depression improvement and the moderating effect of pain severity and pain interference. Growth curve and multivariable regression models were used to test longitudinal associations.

**Results:**

Participants walked an average of 8269 steps per day, and each additional +1000-step difference between participants was significantly associated with a 2.66-point greater improvement (reduction) in BDI-II, controlling for anxiety, pain interference, and adherence to Fitbit monitoring (*P*=.02). Pain severity appeared to moderate (reduce) the positive effect of average daily steps on BDI-II improvement (*P*=.03). Higher baseline depression and anxiety symptoms predicted less positive step trajectories throughout treatment (*P*s≤.001), and more positive step trajectories early in the trial predicted greater MDD improvement at the end of the trial (*P*s<.04). However, step trajectories across the full duration of the trial did not significantly predict MDD improvement (*P*s=.40).

**Conclusions:**

This study used objective measurements to demonstrate positive associations between PA and depression improvement in the context of cognitive behavioral treatment. Pain appeared to moderate this relationship, and baseline symptoms of anxiety and depression predicted PA trajectories. The findings inform future interventions for major depression. Future research with larger samples should consider additional moderators of PA-related treatment success and the extent to which outcomes are related to PA change in multimodal interventions.

**Trial Registration:**

Clinical Trials.gov NCT03406052; https://www.clinicaltrials.gov/ct2/show/NCT03406052

**International Registered Report Identifier (IRRID):**

RR2-10.2196/11591

## Introduction

### Background

Depression is now the leading cause of global disability, severely impacting daily functioning and quality of life for 322 million people worldwide [1]. A significant subset of patients do not respond to antidepressants [2,3] or psychotherapy [4], and about 40% of patients experience relapse following psychotherapeutic treatment [5]. Given the burden of depression, there is a growing emphasis on developing cost-effective, complementary interventions that improve treatment response and reduce relapse risk.

Current evidence supports increased physical activity (PA) and its exercise subsets [6,7] as an adjunctive treatment for major depressive disorder (MDD) or as a single-modality treatment [8-12] with small to moderate antidepressant effects [13,14] and protective benefits against future depression [15]. Despite promising findings, significant research gaps remain.

First, studies investigating the antidepressant effects of PA in patients with MDD have generally relied on self-reported measures [16], which are prone to cognitive biases [17,18] and discrepant results when compared to objectively measured PA [19,20]. Such biases are important to rule out in MDD samples, who often present cognitive impairments [21,22] that can limit the accuracy of self-reported data. Vancampfort et al [23], for example, suggested that individuals with severe mental illness may underestimate their levels of sedentary PA while overestimating vigorous activity, which is consistent with other meta-analytic findings focused on MDD samples [16]. Moreover, Choi et al [19] found that PA was predictive of lower MDD risk when objectively measured but not when subjectively measured. An additional limitation is that many studies that investigated the relationship between depression and objectively measured PA [24] used brief monitoring periods (often 7 days) and samples with specific health conditions that limit the generalizability of findings to patients with MDD.

Another research gap is the lack of data on whether PA’s benefits in MDD samples are influenced by chronic pain. This empirical gap is surprising given the high comorbidity prevalence between pain and depression [25] and findings of pain’s variable effects on PA-treatment outcomes (eg, depression and mental health improvement) [26]. Specifically, PA in pain-free populations often results in reduced pain sensitivity, whereas the relationship is variable in chronic pain populations, with unchanged pain levels in some patients and worsened pain sensitivity in others [27,28]. Thus, the question of whether pain modifies the relationship between PA and depression improvement requires further investigation. Indeed, the heterogeneity of depression has led to calls for more research on moderators of PA’s antidepressant effects [29,30].

A further gap in the empirical literature is that prospective studies typically examine the effect of PA on depressive symptoms [31] without focusing on questions of reverse causality, such as whether baseline depression and anxiety levels predict longitudinal PA patterns [32]. Some studies have found that higher baseline symptoms predict reduced PA over time [33-38], while other studies have not supported this reverse relationship [39,40]. Furthermore, these studies were limited by a reliance on self-reported PA.

Although psychosocial interventions often improve depressive symptoms [41], remarkably little is known about whether positive changes in PA predict such treatment-related improvements [42]. A study found increased PA levels in patients with MDD who responded to repeated transcranial magnetic stimulation [43], and another study found similar associations in patients with cardiovascular disease treated with cognitive behavioral therapy (CBT) [44]. Yet, no previous study has investigated whether objectively measured increases in PA predict MDD symptom reductions in the context of a multimodal mindfulness-based cognitive behavioral therapy (MCBT) intervention for clinical depression. Moreover, it is unclear whether early changes in PA predict future outcomes, despite the clinical importance of identifying early predictors of depression treatment response [45,46], and several studies showing that early increases in behavioral activation predict later symptom improvement [47,48].

### Purpose

This study examined whether daily step counts were associated with MDD symptom improvement over the course of a 24-week internet-based, mindfulness-based cognitive behavioral therapy (iMCBT) intervention for young adults (18-30 years old), controlling for potential confounders (adherence [49,50], baseline anxiety [51,52], and baseline pain [26,53] were selected as covariates given their established relationships with PA and depression improvement). We also examined the interaction effect between steps and baseline pain (severity and interference) on MDD symptom improvement.

A further aim was to examine whether baseline depression and anxiety symptoms were predictive of step trajectories across the trial, and whether step trajectories were associated with MDD symptom improvement. Understanding patterns and moderators of objectively measured PA in relation to treatment outcomes could improve the recommendations for future patients with MDD receiving internet-based cognitive behavioral treatment.

### Hypotheses

Hypothesis 1: Higher average step levels (daily steps) are positively associated with MDD symptom improvement.

Hypothesis 2: Pain severity and pain interference moderate the positive relationship between daily steps and MDD symptom improvement.

Hypothesis 3: Baseline depression and anxiety levels predict week-to-week changes in PA (ie, step trajectories) over the course of iMCBT.

Hypothesis 4: Early step increases and step trajectories across the full length of the iMCBT intervention are positively associated with MDD symptom improvement.

## Methods

### Design and Participants

This secondary analysis uses participant data from the intervention arm of a parallel, 2-arm randomized controlled trial (RCT) comparing iMCBT plus standard psychiatric care (intervention) with standard psychiatric care alone (waitlist control). This analysis did not include the control arm because PA monitoring was part of the intervention arm only. The RCT evaluated the efficacy of an internet- and telephone-based iMCBT intervention for young adults (aged 18-30 years) diagnosed with major depressive disorder. The RCT design [54] and RCT results [55] have been described elsewhere.

All participants were diagnosed by a Centre for Addiction and Mental Health (CAMH) psychiatrist, with diagnoses confirmed through a Mini-International Neuropsychiatric Interview (MINI) administered at the screening visit. Participants were identified from CAMH service waitlists by research coordinators and in the prescreening of new clinic referrals. All self-report measures and clinical interviews were conducted at the same CAMH Ambulatory Service setting. The inclusion and exclusion criteria for this study are listed in Textbox 1.

**Inclusion and exclusion criteria.**

**Inclusion criteria**

- adults aged between 18 and 30 years
- a Beck Depression Inventory-II (BDI-II) score of at least mild severity with no upper limit (BDI-II score ≥14 [56])
- a Mini-International Neuropsychiatric Interview–confirmed psychiatric diagnosis of major depressive disorder [57]
- English fluency

**Exclusion criteria**

- currently receiving weekly structured psychotherapy
- meets the DSM-5 (Diagnostic and Statistical Manual of Mental Disorders, Fifth Edition) criteria for severe alcohol or substance use disorder in the past 3 months
- demonstrates clinically significant suicidal ideation, defined as imminent intent
- attempted suicide in the past 6 months
- diagnosed with borderline personality disorder, bipolar disorder, schizophrenia, or obsessive-compulsive disorder

### Intervention

All RCT participants received standard psychiatric care, defined as monthly treatment-as-usual sessions with a CAMH psychiatrist focused primarily on medication adjustment. Participants in the iMCBT intervention also received a Fitbit-HR Charge 2 (Fitbit Inc) and access to NexJ Connected Wellness (NexJ Health Inc), a cloud-based digital health platform accessible through smartphones and internet-connected devices. Participants were instructed to wear their Fitbit monitors 24 hours daily. Fitbit-tracked daily step counts were automatically uploaded to the NexJ Connected Wellness (NexJ Health Inc) platform, allowing participants and Health Navigator-Coaches to review daily step activity. The purpose of step monitoring was to reinforce iMCBT concepts by providing participants real-time feedback about how the behaviors they modify link to cognitive-affective changes.

The platform also enabled text-message communications between participants and Health Navigator-Coaches, and access to iMCBT content delivered through 24 workbooks and 56 instructional videos reflecting CBT and mindfulness principles. Intervention participants additionally received weekly phone support from Health Navigator-Coaches to facilitate behavior change, the application of iMCBT content, and participant adherence. The workbook content was built on previous web-based MCBT RCTs [58,59] and included a spectrum of MDD-targeted topics such as Living by Your Truths, Overcoming Wired-ness and Tired-ness, Mindfulness and Relationships, Loss and Grief, Resilience, Befriending Ourselves, Befriending Your Body With Exercise, Body Image and Mindfulness, Intimacy, Forgiveness, Overcoming Procrastination, Dealing With Negative Moods, Stress Resilience, Overcoming Performance Anxiety, and Cultivating Inspiration.

### Measures

#### Depression

Depression symptoms at baseline and at the end of the 24-week intervention were measured using the Beck Depression Inventory-II (BDI-II) [60]. The BDI-II is a 21-item self-report inventory for assessing depression symptom severity; each item is measured on a 4-point Likert scale (0 to 3). Higher scores indicate higher levels of depression. The maximum total score is 63. Generally accepted categorical classes are “minimal depression” (0-13), “mild depression” (14-19), “moderate depression” (20-28), and “severe depression” (29-63). The BDI-II has demonstrated acceptable test-retest reliability, convergent and divergent validity [61], and in this sample, acceptable internal consistency (Cronbach α_baseline_=.83, Cronbach α_follow-up_=.90).

#### Anxiety

Baseline anxiety symptoms were measured using the Beck Anxiety Inventory (BAI) [62]. The BAI is a 21-item self-report scale for assessing anxiety severity; each item is measured on a 4-point Likert scale (0 to 3). Higher scores indicate higher levels of anxiety. The maximum total score is 63. The clinical classifications of scored results are “minimal anxiety” (0-7), “mild anxiety” (8-15), “moderate anxiety” (16-25), and “severe anxiety” (26-63). The BAI has demonstrated acceptable test-retest reliability [63], convergent validity [63], and divergent validity [64], and in this sample, acceptable internal consistency (Cronbach α=.78).

#### Pain

Baseline pain dimensions (severity and interference) were measured using the Brief Pain Inventory (BPI) subscales [65]. The BPI is a self-reported measure that gives 2 composite scores representing the severity of pain (Brief Pain Inventory: Severity [BPI-sev]) and the degree to which pain interferes with daily activities (Brief Pain Inventory: Interference [BPI-intf]). The BPI-sev and BPI-intf subscales consist of 4 and 7 items, respectively, and are scored as the average of their respective items. Each item is rated from 0 to 10, with higher ratings indicating worse severity or interference. The maximum composite score for each subscale is 10. The BPI subscales have demonstrated acceptable test-retest reliability and construct validity [66], and in this sample, acceptable internal consistency (Cronbach α_BPI-sev_=.67, Cronbach α_BPI-intf_=.94).

#### Daily Step Count

Physical steps throughout the trial were measured and automatically synchronized with the NexJ Connected Wellness (NexJ Health Inc) platform using the Fitbit-HR Charge 2 (Fitbit Inc), which has demonstrated accurate step estimates in free-living conditions [67-69]. Step counts are a well-established measure of PA, with evidence generally indicating that more is better [70] up to around 8000–10,000 steps/day for adults aged 60 years or younger [71]. Day-to-day step records were obtained from the NexJ Connected Wellness (NexJ Health Inc) platform.

### Statistical Analyses

BDI-II change was operationalized as BDI-II at baseline minus BDI-II at postintervention; higher BDI-II change scores represent greater reductions in depression symptoms (ie, improvement). Daily steps were operationalized as 
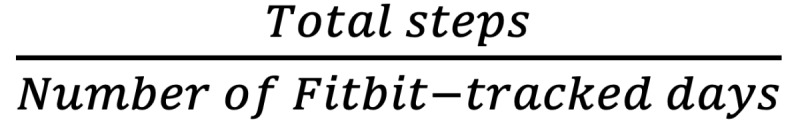
 and adherence to behavioral monitoring was operationalized as 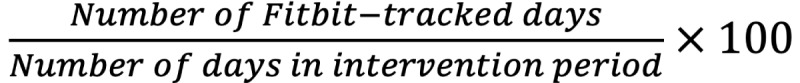.

Step records with fewer than 100 steps were assumed invalid and excluded from the analyses.

Linear regression assumptions were evaluated using graphical methods. Linearity and homoscedasticity were checked using residual plots, and normality was checked using QQ-plots and histograms of model residuals. Hat values, Studentized residuals, and Cook’s distances were used to verify the lack of problematic outliers.

#### Hypothesis 1: Daily Steps Are Positively Associated With MDD Symptom Reduction

Linear regression models were used to examine the effect of daily steps (focal predictor) on BDI-II improvement (outcome), controlling for baseline adherence, anxiety, and pain (severity and interference).

#### Hypothesis 2: Pain Moderates the Relationship Between Steps and BDI-II Improvement

Given the potential for pain to alter the antidepressant effects of daily PA, as well as the observed bimodality in the distribution of BPI subscales (suggestive of 2 clinical subgroups; Figure S1 in Multimedia Appendix 1), we investigated whether pain and daily steps interact to influence BDI-II improvement.

Linear regression was used to evaluate the interaction effects of daily steps × pain interference and daily steps × pain severity on BDI-II improvement.

#### Hypothesis 3: Baseline Depression and Anxiety Levels Predict Week-to-Week Step Trajectories Over the Course of iMCBT

We used 2-level linear conditional growth curve models to test whether baseline characteristics (depression and anxiety) predicted week-by-week step trajectories. To facilitate longitudinal analyses for this hypothesis (and for Hypothesis 4), each participant’s day-to-day steps were collapsed into weekly averages; for example:



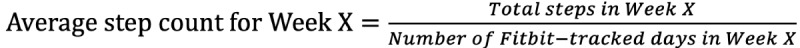



Weekly averages (level 1) were nested within individuals (level 2), and baseline characteristics were treated as level 2 variables. Linear models were considered adequate based on inspection of individual trajectories. Slopes and intercepts were allowed to vary randomly across participants to model change trajectories at the individual level. A first-order autoregressive covariance structure was fit to account for the autocorrelation inherent in longitudinal data. Parameters were estimated using restricted maximum likelihood.

#### Hypothesis 4: Early Step Trajectories (Slopes) and Trajectories Across the Trial Are Positively Associated With Greater BDI-II Improvement

As “early change” has not been consensually operationalized in the psychotherapeutic literature, we defined the first 6 weeks (the first quarter of the iMCBT intervention) as the period of early PA change. To assess whether early step trajectories (slopes) predict later MDD symptom improvement, we first estimated each individual’s slope (
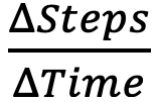
) and intercept (where the slope crosses the y-axis) using week-by-week step averages for the first 6 weeks; the intercept represents the estimated step level at week 0 (ie, at baseline). We then regressed BDI-II change (outcome) on estimated slopes (focal predictor), controlling for intercepts, anxiety, and pain (severity and interference). Intercepts (estimated baseline step levels) were included as a covariate, as past PA behavior is known to predict subsequent PA [72,73] and the development of depressive symptoms [15,31].

To assess whether positive trajectories across the trial were associated with greater MDD symptom reduction, we repeated this procedure using week-by-week step averages for the full duration of the trial. Participants with less than 50% adherence were excluded from the full-length trajectory analysis, as large quantities of missing data can distort slopes and misrepresent change trajectories.

#### Statistical Software

Data cleaning and preparation were carried out using Python (Python Software Foundation). R version 4.1.3 (R Core Team) along with the *nlme* package [74] were used to carry out statistical analyses and mixed-effects modeling. Hypotheses were tested using 2-tailed *t* tests. The significance threshold across analyses was *P*=.05.

### Sample Size Considerations

This study is a secondary analysis of an RCT [55] that originally aimed to enroll 168 participants, with 50% of the participants from a First Nations background and the other 50% from all other ethnic backgrounds, stratified into 2 intervention groups and 2 waitlist control groups (ie, n=42 per group). However, participant enrollment was reduced due to the reluctance of individuals from First Nations backgrounds to participate, despite extensive recruitment efforts. Additionally, given that each recruited participant had to undergo an extensive psychiatric exam to establish a MDD diagnosis, we confronted a limit to the pace of psychiatric examinations that could be scheduled given the existing staff of psychiatrists.

As this is a secondary analysis, no formal power calculations were performed. This approach adheres to the International Council for Harmonisation E9 statistical principles for clinical trials, which state that sample size should be “determined by the primary objective of the trial” [75].

### Ethical Considerations

Ethics approval was obtained from the Research and Ethics Boards of the Centre for Addiction and Mental Health (protocol 115/2016-01) and York University (certificate 2017–154) in Toronto, Ontario, Canada (ClinicalTrials.gov ID NCT03406052). All participants provided in-person written consent for the use of their data in primary and secondary analyses. Participant confidentiality was maintained throughout the study through careful deidentification of data.

## Results

### Overview

A total of 22 participants recruited from February 2018 to September 2018 were enrolled in the iMCBT intervention. Of the 22 iMCBT participants, a total of 2 withdrew due to stressful life events. This secondary analysis is based on the 20 participants who completed the iMCBT intervention. Multimedia Appendix 2 summarizes the flow of participants, and Table 1 shows the sample characteristics for the 20 participants who completed the iMCBT intervention.

**Table 1:** Demographic, psychological, and intervention variables (N=20).

Variable		Value
Age (years), mean (SD)		24.75 (3.48)
**Gender, n (%)**		
	Male	9 (45)
	Female	11 (55)
**Ethnicity, n (%)**		
	Caucasian	12 (60)
	Asian	6 (30)
	Indo-Caribbean	1 (5)
	Mixed	1 (5)
**Education level, n (%)**		
	High school	2 (10)
	Completing college	3 (15)
	College	1 (5)
	Completing university	5 (25)
	University	8 (40)
	Graduate or professional school	1 (5)
**Work status, n (%)**		
	Employed	11 (55)
	Unemployed	9 (45)
**Depression duration, mean (SD)**		
	Age of depression onset (years)	17.20 (4.10)
	Number of depressive episodes in lifetime (n=19)	5.16 (5.10)
	Duration of current depressive episode (months)	8.88 (15.30)
**Psychological variables at baseline, mean (SD)**		
	BDI-II^a^	29.20 (8.22)
	BAI^b^	28.15 (8.22)
	BPI-sev^c^	1.69 (1.46)
	BPI-intf^d^	1.99 (2.55)
**Outcome and intervention variables, mean (SD)**		
	BDI-II improvement^e^	15.60 (9.75)
	Daily steps^f^	8263 (2842)
	Total steps	1,196,806 (678,338)
	Adherence^g^ (percentage), median (IQR)	84.35 (70.08-98.36)

^a^BDI-II: Beck Depression Inventory-II.

^b^BAI: Beck Anxiety Inventory.

^c^BPI-sev: Brief Pain Inventory: Severity.

^d^BPI-intf: Brief Pain Inventory: Interference.

^e^Pretreatment BDI-II minus posttreatment BDI-II.

^f^Total steps recorded divided by number of Fitbit-tracked days.

^g^Number of Fitbit-tracked days divided by number of days in intervention period.

### Hypothesis 1: Daily Steps Are Positively Associated With MDD Symptom Reduction

Multivariable regression was used to test the relationship between daily steps and BDI-II reduction, controlling for potential confounders. The daily steps variable was rescaled by a divisor of 1000 to improve the interpretability of parameter estimates. Results are presented in Table 2.

Per Model A, there was evidence of a nonsignificant positive relationship between daily steps and BDI-II improvement, controlling for adherence, pain severity, and anxiety (*b*=1.95; SE=0.98; *t*_15_=1.99; *P*=.06). In Model B, where we controlled for pain interference instead of pain severity, there was a significant positive effect of daily steps on BDI-II improvement (*b*=2.66; SE=1.00; *t*_15_=2.65; *P*=.02).

**Table 2:** Regression of Beck Depression Inventory-II (BDI-II) change on steps, adherence, and comorbidities (N=20).

Variable		*b*	SE	*t* (*df*)	*P* value	95% CI	*R* ^2^
**Model A**							.42^a^
	(Intercept)	–44.08	21.23	–2.08 (15)	.06	–89.32 to 1.17	
	Daily steps (per 1000)	1.95	0.98	1.99 (15)	.06	–0.13 to 4.03	
	Adherence	0.25	0.10	2.60 (15)	.02	0.04 to 0.45	
	BPI-sev^b^	3.94	1.68	2.35 (15)	.03	0.36 to 7.52	
	BAI^c^	0.62	0.36	1.72 (15)	.11	–0.15 to 1.40	
**Model B**							.49^d^
	(Intercept)	–43.76	19.51	–2.24 (15)	.04	–85.34 to –2.17	
	Daily steps (per 1000)	2.66	1.00	2.65 (15)	.02	0.52 to 4.79	
	Adherence	0.15	0.09	1.72 (15)	.11	–0.04 to 0.33	
	BPI-intf^e^	2.55	0.88	2.91 (15)	.01	0.68 to 4.42	
	BAI	0.74	0.34	2.17 (15)	.05	0.01 to 1.46	

^a^*F*_4,15_=2.68; *P*=.07.

^b^BPI-sev: Brief Pain Inventory: Severity.

^c^BAI: Beck Anxiety Inventory.

^d^*F*_4,15_=3.61; *P*=.03.

^e^BPI-intf: Brief Pain Inventory: Interference.

### Hypothesis 2: Pain Moderates the Relationship Between Steps and BDI-II Improvement

There was evidence of a significant interaction between daily steps and BPI-sev: *b*_(Steps × BPI-sev)_=–1.45; SE=0.62; *t*_16_=–2.36; *P*=.03. The interaction effect explained an additional 22.21% of the variance in BDI-II improvement (Δ*R*^2^=.22) over the variance explained by the additive effects of daily steps and BPI-sev (for the additive model, see Table S1 in Multimedia Appendix 1).

By contrast, the interaction between daily steps and BPI-intf was not significant: *b*_(Steps × BPI-intf)_=–0.61; SE=0.38; *t*_16_=–1.59; *P*=.13. The interaction effect explained an additional 9.45% of the variance in BDI-II improvement (Δ*R*^2^=.09) over the variance explained by the additive effects of daily steps and BPI-intf (for the additive model, see Table S1 in Multimedia Appendix 1).

**Figure 1:**
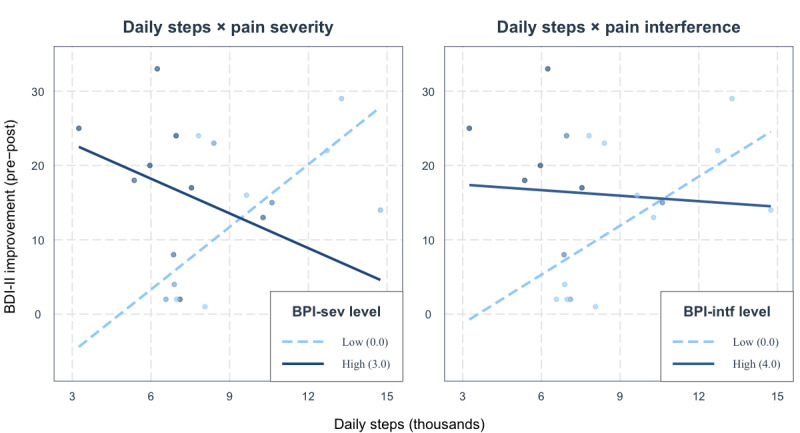
Visualization of the effect of steps on Beck Depression Inventory-II (BDI-II) improvement at high and low levels of pain severity and pain interference (N=20). At low levels of pain, more physical activity in the form of daily steps was associated with greater symptom improvement, whereas this positive association tended to weaken as pain levels increased. BPI-intf: Brief Pain Inventory: Interference; BPI-sev: Brief Pain Inventory: Severity.

Table 3 summarizes the regression results, Figure 1 depicts the interaction, and Table 4 describes the conditional effect of daily steps on BDI-II improvement at low (25th percentile) and high (75th percentile) levels of BPI-sev and BPI-intf.

Overall, the effect of daily steps on MDD symptom improvement was positive at low levels of pain, but this effect tended to decrease as pain increased. Pain therefore appears to modify the antidepressant effect of steps.

**Table 3:** Regression of Beck Depression Inventory-II (BDI-II) improvement on steps, pain, and their interaction (N=20).

Variable		*b*	SE	*t* (*df*)	*P* value	95% CI	*R* ^2^
**Model A**							.36^a^
	(Intercept)	–13.52	10.73	–1.26 (16)	.23	–36.26 to 9.22	
	Daily steps (per 1000)	2.80	1.08	2.60 (16)	.02	0.52 to 5.09	
	BPI-sev^b^	13.70	4.84	2.83 (16)	.01	3.44 to 23.96	
	Daily steps × BPI-sev	–1.45	0.62	–2.36 (16)	.03	–2.76 to –0.15	
**Model B**							.41^c^
	(Intercept)	–7.88	8.59	–0.92 (16)	.37	–26.08 to 10.32	
	Daily steps (per 1000)	2.20	0.88	2.50 (16)	.02	0.34 to 4.06	
	BPI-intf^d^	6.51	2.64	2.47 (16)	.03	0.92 to 12.11	
	Daily steps × BPI-intf	–0.61	0.38	–1.59 (16)	.13	–1.43 to 0.20	

^a^*F*_3,16_=3.05; *P*=.06.

^b^BPI-sev: Brief Pain Inventory: Severity.

^c^*F*_3,16_=3.63; *P*=.04.

^d^BPI-intf: Brief Pain Inventory: Interference.

**Table 4:** Conditional effect of daily steps on Beck Depression Inventory-II (BDI-II) improvement at different levels of pain (N=20).

Variable		*b*_Steps_	SE	*t* (*df*)	*P* value	95% CI
**Pain severity level (BPI-sev)^a^**						
	0 (Low)	2.81	1.08	2.60 (16)	.02	0.52 to 5.09
	3 (High)	–1.56	1.40	–1.11 (16)	.28	–4.53 to 1.41
**Pain interference level (BPI-intf)^b^**						
	0 (Low)	2.20	0.88	2.50 (16)	.02	0.34 to 4.06
	4 (High)	–0.25	1.44	–0.17 (16)	.87	–3.29 to 2.80

^a^BPI-sev: Brief Pain Inventory: Severity.

^b^BPI-intf: Brief Pain Inventory: Interference.

### Hypothesis 3: Baseline Depression and Anxiety Levels Predict Week-To-Week Step Trajectories Over the Course of iMCBT

Conditional growth models were used to test whether changes in steps over time (trajectories) varied as a function of baseline depression or anxiety. We use *b*_Time_ to denote the effect of an additional week on the weekly average of steps, conditioned on the levels of baseline depression or baseline anxiety.

**Figure 2:**
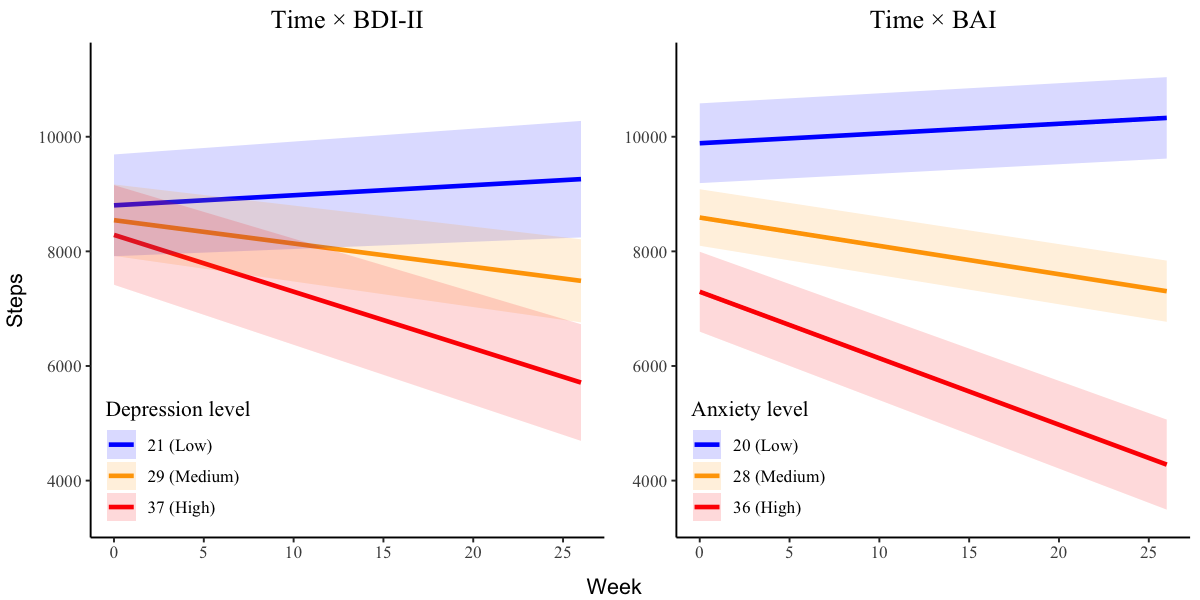
Visualization of step trajectories over the course of internet-based, mindfulness-based cognitive behavioral therapy (iMCBT) at different levels of baseline depression and anxiety (N=20). Higher baseline levels of depression and anxiety were associated with larger decreases in physical activity over the course of treatment. BAI: Beck Anxiety Inventory; BDI-II: Beck Depression Inventory-II.

Depression (*b*_Time × BDI-II_=–7.29) and anxiety (*b*_Time × BAI_=–8.32) were significant moderators of step trajectories (Table 5), indicating that greater baseline BDI-II or BAI severity was associated with larger step reductions over the course of the intervention. Figure 2 depicts the interaction effects and Table 6 provides the corresponding trajectory estimates (*b*_Time_) at low, medium, and high levels of BDI-II and BAI (levels calculated as mean ± SD).

**Table 5:** Results from longitudinal modeling of estimated step trajectories conditioned on baseline depression or baseline anxiety (N=20).

Variable		*b*	SE	*t* (*df*)	*P* value	95% CI
**Model BDI-II^a^**						
	(Intercept)	9479.56	2344.89	4.04 (417)	<.001	4870.27 to 14,088.85
	Time (Week)	170.77	67.11	2.54 (417)	.01	38.86 to 302.69
	BDI-II	–32.22	77.50	–0.42 (18)	.68	–195.03 to 130.59
	Time × BDI-II	–7.29	2.27	–3.21 (417)	.001	–11.76 to –2.83
**Model BAI^b^**						
	(Intercept)	13124.06	1788.53	7.34 (417)	<.001	9608.40 to 16,639.72
	Time (Week)	183.48	60.48	3.03 (417)	.003	64.59 to 302.37
	BAI	–161.87	61.43	–2.64 (18)	.02	–290.92 to –32.82
	Time × BAI	–8.32	2.21	–3.77 (417)	<.001	–12.66 to –3.98

^a^BDI-II: Beck Depression Inventory-II.

^b^BAI: Beck Anxiety Inventory.

**Table 6:** Step trajectories (slopes) at different levels of baseline depression and anxiety (N=20).

Variable		*b*_Time_	SE	*t* (*df*)	*P* value	95% CI
**Depression level (BDI-II)^a^**						
	21 (Low)	17.60	25.11	0.70 (417)	.48	–31.77 to 66.97
	29 (Medium)	–40.75	18.80	–2.17 (417)	.03	–77.70 to –3.80
	37 (High)	–99.10	27.15	–3.65 (417)	<.001	–152.47 to –45.74
**Anxiety level (BAI)^b^**						
	20 (Low)	17.06	22.40	0.76 (417)	.45	–26.98 to 61.10
	28 (Medium)	–49.51	18.23	–2.72 (417)	.007	–85.34 to –13.67
	36 (High)	–116.08	28.06	–4.14 (417)	<.001	–171.23 to –60.92

^a^BDI-II: Beck Depression Inventory-II.

^b^BAI: Beck Anxiety Inventory.

### Hypothesis 4: Early Step Trajectories (Slopes) and Trajectories Across the Trial Are Positively Associated With Greater BDI-II Improvement

#### Early Step Trajectories and BDI-II Improvement

Regression models were used to test the relationship between early step increases (slopes) and BDI-II improvement, controlling for intercepts (estimated step levels at week 0), pain, and anxiety.

“Early” was defined as the first 6 weeks of iMCBT. Slopes represent the linear change in weekly step averages per week (eg, a slope estimate of 20 would indicate that each week, the weekly step average increased by 20). Thus, a 1-unit increase in an individual’s slope would mean 1 additional average step per week. The average slope during the first 6 weeks of iMCBT was –35.47 (SD 403.73), and the average initial step level (intercept) was 8467.74 (SD 2847.34).

Per Table 7, the effect of slopes on BDI-II improvement was significant, controlling for estimated initial step levels, BPI-sev, and BAI (*b*=0.0137; SE=0.0060; *t*_15_=2.26; *P*=.04). The effect of slopes was also significant when controlling for BPI-intf and the other 2 covariates (*b*=0.0165; SE=0.0051; *t*_15_=3.23; *P*=.006).

In other words, each 100-unit increase (per week) in the weekly average of steps during early iMCBT predicted a 1.65 greater BDI-II improvement at the end of iMCBT, controlling for pain interference and covariates. The effect of early step increases was slightly weaker when controlling for pain severity and covariates.

**Table 7:** Regression of Beck Depression Inventory-II (BDI-II) change on 6-week step trajectories, adjusted for initial step levels, anxiety, and pain (N=20).

Variable		*b*	SE	*t* (df)	*P* value	95% CI	*R* ^2^
**Model A**							.30^a^
	(Intercept)	3.49	14.17	0.25 (15)	.81	–26.71 to 33.70	
	Slope	0.0137	0.0060	2.26 (15)	.04	0.0008 to 0.0265	
	Step level (per 1000; week 0)	1.05	0.98	1.08 (15)	.30	–1.04 to 3.14	
	BPI-sev^b^	3.20	1.81	1.77 (15)	.10	–0.65 to 7.04	
	BAI^c^	–0.06	0.31	–0.20 (15)	.85	–0.73 to 0.61	
**Model B**							.52^d^
	(Intercept)	–14.93	13.47	–1.11 (15)	.29	–43.65 to 13.79	
	Slope	0.0165	0.0051	3.23 (15)	.006	0.0056 to 0.0274	
	Step level (per 1000; week 0)	2.41	0.96	2.51 (15)	.02	0.36 to 4.46	
	BPI-intf^e^	3.02	0.90	3.36 (15)	.004	1.10 to 4.94	
	BAI	0.17	0.24	0.68 (15)	.51	–0.35 to 0.68	

^a^*F*_4,15_=1.64; *P*=.22.

^b^BPI-sev: Brief Pain Inventory: Severity.

^c^BAI: Beck Anxiety Inventory.

^d^*F*_4,15_=4.06; *P*=.02.

^e^BPI-intf: Brief Pain Inventory: Interference.

#### Step Trajectories Across iMCBT and BDI-II Improvement

BDI-II change was regressed on slopes across the full duration of iMCBT, controlling for covariates. A total of 3 participants were excluded due to adherence levels below 50%. The average slope across the entire trial was –20.68 (SD 85.74), and the average initial step level (intercept) was 8492.71 (SD 2887.52). Slopes across the trial were not significantly associated with MDD symptom improvement, controlling for

- initial step levels and BAI, *b*=0.0430; *P*=.40; 95% CI (–0.0635 to 0.1495)
- initial step levels and BPI-sev, *b*=–0.0223; *P*=.40; 95% CI (–0.0779 to 0.0332)
- initial step levels and BPI-intf, *b*=–0.0208; *P*=.40; 95% CI (–0.0728 to 0.0313)

Full regression results are presented in Table S2 in Multimedia Appendix 1.

## Discussion

This study examined daily steps in relation to MDD symptom improvement and baseline characteristics in a youth sample who had completed a 24-week iMCBT treatment for mild to severe depression.

### Principal Findings

The average patient in our sample was moderately to severely depressed at baseline (mean 29.20, SD 8.22). Over the course of treatment, participants took an average of 8269 steps per day, within the suggested optimal dose of 8000-10,000 [71,76]. We found that each additional 1000-step increment was significantly associated with a 2.66-point reduction in MDD symptoms (BDI-II) after controlling for adherence, anxiety, and pain interference. This finding is consistent with research showing that PA dose positively predicts greater antidepressant response [77]. The effect has clinical relevance given that in our sample, a 5-point reduction is considered a clinically meaningful improvement (based on a 17.5% reduction in BDI-II from baseline [78]). Thus, an additional 2000 steps daily would be associated with a meaningful reduction in depression symptoms. When we controlled for pain severity instead of pain interference, the 1000-step effect was of similar magnitude (*b*=1.95) but nonsignificant.

Given the observed bimodality in our sample’s distribution of pain scores and previous evidence of pain’s differential effects on PA-related benefits [28], we explored the interaction effect between pain (severity and interference) and daily steps on MDD symptom reduction. Analyses showed that as pain levels increased, the effect of steps on depression symptom reduction decreased. Our finding is consistent with previous work demonstrating pain’s negative impact on depression treatment response [79-81], and suggests that while PA is consistently supported in the management of pain conditions [82], PA prescriptions for pain should be tailored to individual circumstances for optimal response [83-86]. In patients with fibromyalgia, for example, low to moderate PA is often associated with successful outcomes [87,88] whereas strenuous exercise induces hyperalgesia [28,89]. Additionally, dysfunction in stress response systems is often observed in patients with chronic pain [90], resulting in increased vulnerability to PA-related stress [84]. Thus, the smaller symptom reductions associated with excess PA among patients with higher pain levels may reflect impaired stress adaptation and inadequate restoration of homeostatic balance [84,91]. Future research is needed to verify these findings in larger, more diverse samples.

Additional study findings suggest that higher baseline levels of depression and anxiety were associated with larger decreases in PA over the course of iMCBT treatment. These results are consistent with other prospective research showing that initial anxiety and depression severity levels predict reduced PA over time [35,37]. Patients with greater depressive symptoms likely had less energy, confidence, or motivation [92] needed to persist in PAs. Additionally, anxiety disorders are often associated with social withdrawal and avoidance behaviors [93] that favor reduced PA and increased sedentariness [94,95]. Our findings are distinct from previous research in that the present associations occur in the context of a multimodal iMCBT intervention.

There was evidence that early PA increases predicted greater MDD symptom improvement at the end of the trial. Specifically, during the early weeks of iMCBT, each 100-unit increase in the weekly average of steps was associated with a 1.65-point reduction in BDI-II severity, controlling for pain interference, anxiety, and estimated step levels at baseline. Thus, a clinically meaningful reduction in BDI-II of 5 points would be expected if a patient’s weekly step average increased by 300 steps each week during early iMCBT. This finding is consistent with previous evidence demonstrating the predictive value of early changes for later outcomes [96], with most studies focusing on early psychological gains [45,97,98] and fewer on early behavioral gains [47,48]. Our finding extends the literature by adding objectively measured PA to the accumulated evidence of early predictors of final treatment outcomes.

We did not find support for the hypothesis that, across the full duration of iMCBT, positive step trajectories would predict greater depression improvement. This was possibly due to the length of the trial combined with the heterogeneous nature of depression. It is well established that symptoms vary substantially between patients [99,100], with at least 1030 unique symptom profiles identified [101]. Therefore, as iMCBT treatment progressed, therapeutic tasks also progressed with increasing differentiation. For example, increasing approach behaviors may have been emphasized for patients prone to depressive avoidance [102], whereas calming mindfulness techniques may have been more important for patients with anxious distress [103]. Given the multimodal nature of iMCBT, varying therapeutic tasks were emphasized with differing implications for long-term PA trajectories.

### Limitations

This study has several limitations. First, our sample size was modest, but this limitation was balanced against strict sampling criteria and intervention duration. Whether the study findings generalize will be confirmed in a future, larger trial targeting MDD in a broader age group [104].

Second, there was no measure of PA intensity (eg, moderate vs vigorous PA). Step count combined with intensity metrics might have enabled more detailed PA profiles, revealing more diverse impacts on depression.

Additionally, a basic measure like step count does not account for certain PAs (eg, strength training and water-based activities) that may contribute to depression improvement, though trial observations indicate that participants rarely engaged in such activities.

Lastly, while our data do not account for adherence to Fitbit monitoring at the within-day level, patients were instructed to wear their Fitbit monitors 24 hours daily. As an approximation of intraday adherence, we estimated day-to-day adherence (ie, days tracked divided by intervention period), which was included as a covariate in most of the multivariable regression models. To further account for possible missing intraday data, we removed daily step counts less than 100. Although a 100-step threshold may seem low given that previous researchers have used thresholds as high as 1000 [105], we considered potential vegetative symptoms commonly associated with major depression. Further, we evaluated the sensitivity of our results to a 1000-step threshold and found that it yielded only slightly different effect sizes and no substantive changes to inferential tests.

### Conclusions

This is the first known prospective study to investigate objectively measured PA and its associations with depression symptom improvement during a multimodal cognitive-behavioral intervention. Our findings suggest that higher activity levels were associated with larger improvements and that this relationship may be influenced by chronic pain. We also extend the current literature by showing that initial symptom severity predicts PA change throughout treatment, and that early increases in PA predict better final outcomes. Given the role of PA in relapse prevention, it may be valuable to target activity levels in those with heightened symptoms early in the course of treatment. Future research is needed to examine how and to what extent PA changes can maximize treatment response in the context of multimodal interventions.

## Appendix

Multimedia Appendix 1 Supplementary figures and and tables.

Multimedia Appendix 2 CONSORT (Consolidated Standards of Reporting Trials) diagram. iMCBT: internet-based, mindfulness-based cognitive behavioral therapy.

Multimedia Appendix 3 CONSORT-eHEALTH checklist (V 1.6.1).

## Abbreviations

BAI: Beck Anxiety Inventory
BDI-II: Beck Depression Inventory-II
BPI: Brief Pain Inventory
BPI-intf: Brief Pain Inventory: Interference
BPI-sev: Brief Pain Inventory: Severity
CAMH: Centre for Addiction and Mental Health
CBT: cognitive behavioral therapy
DSM-5: Diagnostic and Statistical Manual of Mental Disorders, Fifth Edition
iMCBT: internet-based, mindfulness-based cognitive behavioral therapy
MCBT: mindfulness-based cognitive behavioral therapy
MDD: major depressive disorder
MINI: Mini-International Neuropsychiatric Interview
PA: physical activity
RCT: randomized controlled trial

## Data Availability

The data sets generated during and analyzed during this study are available from the corresponding author on reasonable request.
