# Supplementary material for: The Role of Daily Steps in the Treatment of Major Depressive Disorder: Secondary Analysis of a Randomized Controlled Trial of a 6-Month Internet-Based, Mindfulness-Based Cognitive Behavioral Therapy Intervention for Youth
Source: Interact J Med Res. 2023 Dec 8;12:e46419. doi: 10.2196/46419 (PMC10746981; doi:10.2196/46419)
Supplement: Multimedia Appendix 1 [file ijmr_v12i1e46419_app1.docx]

**Figure S1**. Distributions of Brief Pain Inventory subscales (N=20). Kernel density was estimated using a Gaussian kernel.


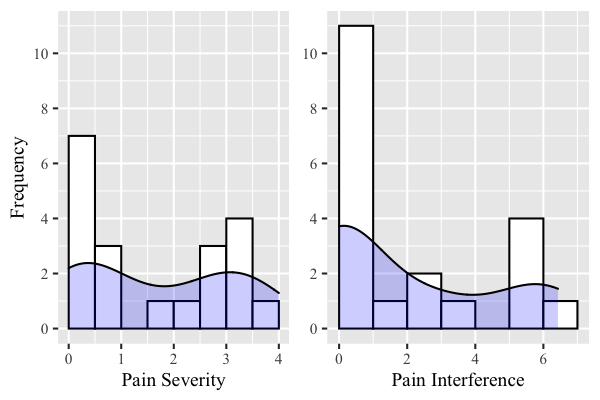


**Table S1**. Additive models of Beck Depression Inventory-II (BDI-II) improvement regressed on steps and pain (N=20).

| Variable | *b* | *SE* | *t*(17) | *P* value | 95% CI | *R*^2^ |
| --- | --- | --- | --- | --- | --- | --- |
| **Additive Model A** | |  |  |  |  | .14^a^ |
| (Intercept) | 1.35 | 9.79 | 0.14 | .89 | [-19.30, 22.00] |  |
| Daily steps (per 1000) | 1.13 | 0.92 | 1.24 | .23 | [-0.80, 3.07] |  |
| BPI-sev | 2.89 | 1.79 | 1.62 | .12 | [-0.88, 6.66] |  |
| **Additive Model B** | |  |  |  |  | .31^b^ |
| (Intercept) | -3.07 | 8.39 | -0.37 | .72 | [-20.78, 14.64] |  |
| Daily steps (per 1000) | 1.65 | 0.84 | 1.95 | .07 | [-0.13, 3.42] |  |
| BPI-intf | 2.56 | 0.94 | 2.73 | .01 | [0.58, 4.54] |  |

*Note*. BPI-sev = Brief Pain Inventory: Severity; BPI-intf = Brief Pain Inventory: Interference.

^a^*F*(2, 17) = 1.40, *P*=.27. ^b^*F*(2, 17) = 3.83, *P*=.04.

**Table S2.** Regression of Beck Depression Inventory-II (BDI-II) improvement on step trajectories across iMCBT, adjusted for initial step levels, anxiety, and pain (n=17).

| Variable | *b* | *SE* | *t*(13) | *P* value | 95% CI | *R*^2^ |
| --- | --- | --- | --- | --- | --- | --- |
| **Model A** |  |  |  |  |  | .13^a^ |
| (Intercept) | -19.33 | 26.26 | -0.74 | .48 | [-76.07, 37.41] |  |
| Slope | 0.0430 | 0.05 | 0.87 | .40 | [-0.0635, 0.1495] |  |
| Step level (per 1000; week 0) | 1.80 | 1.38 | 1.31 | .21 | [-1.17, 4.78] |  |
| BAI | 0.79 | 0.63 | 1.24 | .24 | [-0.58, 2.15] |  |
| **Model B** |  |  |  |  |  | .38^b^ |
| (Intercept) | -13.07 | 11.23 | -1.16 | .27 | [-37.32, 11.18] |  |
| Slope | -0.0223 | 0.03 | -0.87 | .40 | [-0.0779, 0.0332] |  |
| Step level (per 1000; week 0) | 2.41 | 1.02 | 2.36 | .03 | [0.20, 4.61] |  |
| BPI-sev | 5.66 | 2.06 | 2.74 | .02 | [1.20, 10.11] |  |
| **Model C** |  |  |  |  |  | .45^c^ |
| (Intercept) | -11.00 | 9.56 | -1.15 | .27 | [-31.65, 9.65] |  |
| Slope | -0.0208 | 0.02 | -0.86 | .40 | [-0.0728, 0.0313] |  |
| Step level (per 1000; week 0) | 2.37 | 0.92 | 2.57 | .02 | [0.38, 4.36] |  |
| BPI-intf | 3.14 | 1.00 | 3.16 | .008 | [0.99, 5.29] |  |

*Note*. iMCBT = Internet-based, mindfulness-based cognitive-behavioral therapy; BPI-sev = Brief Pain Inventory: Severity; BPI-intf = Brief Pain Inventory: Interference; BAI = Beck Anxiety Inventory.

^a^*F*(3, 13) = 0.63, *P*=.61. ^b^*F*(3, 13) = 2.68, *P*=.09. ^c^*F*(3, 13) = 3.51, *P*=.046.
