# Supplementary figures and images for: The Role of Daily Steps in the Treatment of Major Depressive Disorder: Secondary Analysis of a Randomized Controlled Trial of a 6-Month Internet-Based, Mindfulness-Based Cognitive Behavioral Therapy Intervention for Youth
Source: Interact J Med Res. 2023 Dec 8;12:e46419. doi: 10.2196/46419 (PMC10746981; doi:10.2196/46419)

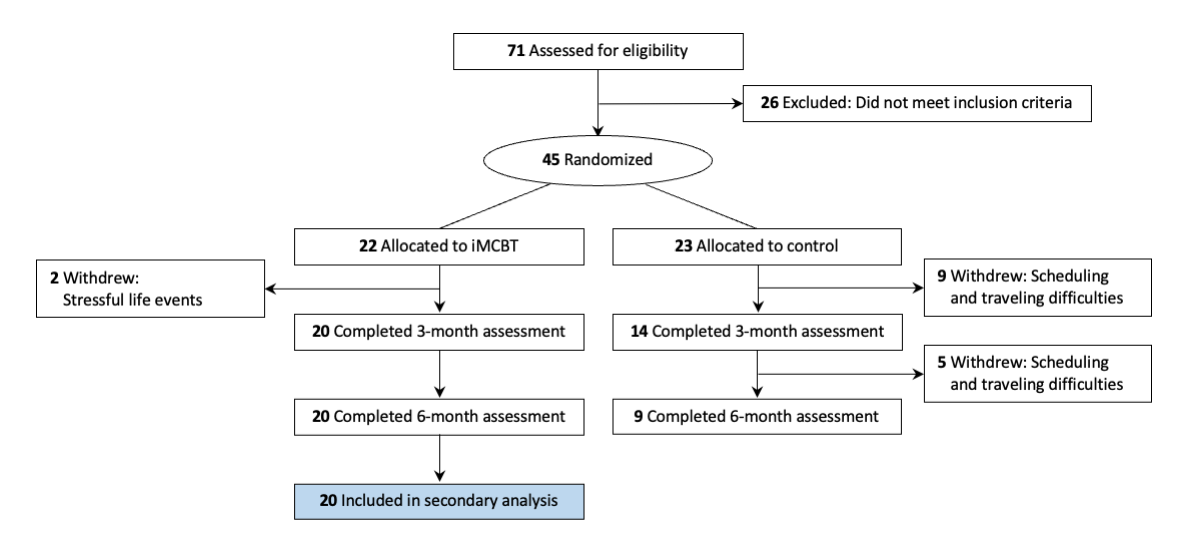

Supplement: Multimedia Appendix 2 [file ijmr_v12i1e46419_app2.png]
